# Examining a technology-focused language teacher community on Facebook during a crisis situation

**DOI:** 10.1186/s40862-022-00159-0

**Published:** 2023-01-03

**Authors:** Yurika Ito

**Affiliations:** School of International Liberal Studies, Waseda University, Tokyo, Japan

**Keywords:** Language teacher learning, Online language teacher communities, Facebook, Social networking sites (SNSs)

## Abstract

Due to the chaos and confusion caused by the sudden transition from face-to-face teaching to online and remote teaching in early 2020, numerous language teachers had no choice but to rely on online communities on social networking sites. The current study therefore examined how some language teachers were utilising online communities on Facebook during the COVID-19 pandemic. Employing a mixed-methods approach, data were mainly collected through: (1) an eight-month observation of a technology-focused language teacher community on Facebook to identify different types of posts generated by its members before and during the COVID-19 pandemic (n = 340); (2) a questionnaire to understand the community members’ backgrounds and experiences of being in the community (n = 51); (3) semi-structured interviews with some of the questionnaire participants (n = 13); and (4) a post-interview questionnaire (n = 12) to get a better understanding of their responses. A content analysis of online posts and community members’ responses suggest that language teacher communities on Facebook were supporting teachers during the stressful periods of the pandemic professionally and emotionally. The main findings are discussed in terms of the benefits and drawbacks of using online language teacher communities for professional purposes. The overall goal of the study is to offer much-needed answers on how pre-existing communities can be used to assist language teachers in times of a crisis.

## Introduction

Many language teachers around the world, including those who were once reluctant to use technology for teaching purposes, were forced to temporarily teach online because of the COVID-19 pandemic (e.g., Cheung, 2021; Marchlik et al., 2021; Moorhouse & Kohnke, 2021). Although the use of technology in language learning classrooms is not a new concept (e.g., Chapelle & Sauro, 2017; Farr & Murray, 2016; Levy & Hubbard, 2005; Stockwell, 2012, 2022), until the pandemic, not all language teachers were concerned with implementing technology into their own classes (Wang, 2021). As a result of the unexpected health crisis in early 2020, teachers were faced with unprecedented urgency to learn how to teach online (Trust et al., 2020). In times of a pandemic, the value of online communities on SNSs is apparent as many teachers were left alone to navigate their “new” virtual language classrooms with little or no training (MacIntyre et al., 2020). Without the need to leave their homes, language teachers are able to connect and interact with other teachers who are also teaching in similar circumstances. Considering that not much actual research has been undertaken so far (cf. Al-Jarf, 2021), this study therefore aims to investigate how some language teachers are making use of online teacher communities on Facebook for professional purposes amidst the pandemic.

## Background

### Enhancing teachers’ professional knowledge and skills

Regardless of the pandemic, for language teachers to implement the best educational practice, they need to continuously advance their professional knowledge and skills throughout their careers. To date, there has been a considerable amount of research concerning teacher professional learning, which can be broadly defined as “learning resulting from any activity that is intended to prepare teachers for improved performance in present or future practice” (Prenger et al., 2019, p. 442). Teacher professional learning opportunities come in a variety of forms, ranging from traditional classroom-based learning to online learning. As top-down approaches to professional learning, such as brief one-time teacher workshops mandated by schools and universities, have often been regarded as ineffective (Farrell, 2022; Whitaker & Valtierra, 2019), there has been a demand for alternative learning opportunities (e.g., Chen et al., 2009; Crandall & Christison, 2016).

For professional learning to be sustained continuously throughout the teachers’ long careers, it is essential for the ways of learning to specifically cater to the teachers’ working conditions and needs (Duncan-Howell, 2010). Considering that teachers generally have a heavy and demanding workload (Williamson & Myhill, 2008), professional learning opportunities need to be provided in a way that fit into their busy schedules. Moreover, since teachers are at different stages of their careers (Eros, 2011), instead of having a one-size-fits-all approach to professional learning, the content should be flexible and meet the individual teachers’ demands. Another important consideration is how the learning experience takes place (Duncan-Howell, 2010). Some teachers prefer learning individually, whereas others prefer learning collaboratively. Whilst both forms have their strengths and weaknesses (Nokes-Malach et al., 2015), there have been some evidence to suggest that collaboration plays a key role in encouraging the learning of teachers (e.g., Jong et al., 2019; Vangrieken et al., 2015). As teacher isolation has long been a widespread issue (e.g., Davis, 1986–1987; Lam & Lau, 2012), collaboration may especially help teachers who are isolated at their workplace. Bearing in mind these factors, online communities, which “promote collaboration among individuals in many different regions or even nations” (Crandall & Christison, 2016, p. 18), seem to suit the needs of a modern-day teacher and have the potential of being a viable option for promoting teacher professional learning, though questions still remain as how they can actually support teachers.

### Teachers’ uses of online communities on SNSs

The development of Collaborative Web technologies (i.e., Web 2.0), which include SNSs (Greenhow et al., 2009), have led to the formation of numerous online communities. Over ten million communities currently exist on Facebook alone (Meta, 2019). Online communities on SNSs are characteristically easy to become a part of as they are often free and can be joined with a push of a button.

Past studies investigating the educational potential of online communities for supporting teachers have explored different SNSs, most typically Facebook (e.g., Bissessar, 2014; Nilimarkka et al., 2021; Patahuddin & Logan, 2018; Rutherford, 2010; Yildirim, 2019), Twitter (e.g., Britt & Paulus, 2016; Carpenter & Krutka, 2015; Curwood & Scott, 2017; Wesely, 2013), Instagram (e.g., Carpenter et al., 2020), Pinterest (e.g., Schroeder et al., 2019), and Reddit (e.g., Staudt Willet & Carpenter, 2020). One of the most cited works on online language teacher communities on SNSs is the study conducted by Wesely (2013) who examined the ways in which language teachers around the world used Twitter for their professional learning. Based on the findings from participant observations of online language teacher communities on Twitter and interviews with nine participating language teachers, the researcher showed how the online language teacher communities on Twitter not only facilitated the learning of the teachers but also helped them overcome the feeling of collegial isolation. The members reported on how being a part of the language communities on Twitter positively affected their teaching and enabled them to create new ties with other teachers with diverse backgrounds.

Despite the dearth of research into Facebook language teacher communities, the literature on the use of Facebook communities by teachers of other subjects can still be of relevance. For instance, through examining posts shared in a Facebook community for mathematics teachers for a period of one month (n = 2442) and semi-structured interviews with some of its members (n = 14), Yildirim (2019) found that members were asking for teaching advice and ideas as well as sharing teaching materials, information about private courses, and information about university entrance examinations and tests. Similarly, in another study, Rutherford (2010) who conducted a content analysis of 187 posts shared in a general teacher community on Facebook found that the members posted messages to seek advice and ideas about how to make their classes more creative, engaging, and interactive.

Whilst these studies demonstrate that online teacher communities on SNSs bring about various benefits to teachers’ professional lives, the negative aspects should also be noted. Not the least of these is the difficult aspect of sustaining a successful online community, as indicated in a recent study conducted by Nilimarkka et al. (2021) who examined eight years-worth of posts, comments, and reactions shared in a technology-focused online teacher community on Facebook. This extensive examination of a large teacher community with nearly 20,000 teachers revealed how only a small proportion of the members were truly active and how it is difficult to facilitate teachers’ learning in communities when there are many members. Moreover, Goodyear et al. (2019) who observed the interactions of physical education teachers on Twitter, reported that some of the observed members hijacked the discussions and made it difficult for members with lower status to participate. It is clear from these two studies alone that online teacher communities on SNSs are not without flaws and that teachers may not necessarily hold positive attitudes towards using them for professional purposes.

### Current literature gaps

As illustrated in the preceding section, there have been an increasing number of studies investigating the general use of online teacher communities in non-language teaching contexts. However, since language teachers have specific needs in that they need to continuously enhance their professional knowledge, including subject knowledge about a language (e.g., grammar, phonology, vocabulary) and pedagogical knowledge of how to teach language skill areas to learners with different backgrounds using a wide variety of tools and materials, throughout their careers (e.g., Farrell, 2022; Richards & Farrell, 2005; Son, 2018), their needs are fundamentally different from teachers teaching other subjects. Despite the need for this special consideration, not many studies have specifically focused on examining how language teacher are interacting with one another in online language teacher communities (cf. Wesely, 2013), and there have been even fewer studies about technology-focused language teacher communities. Hence, although prior studies have outlined some of the key benefits and negative aspects associated with the general use of teacher communities, further exploration is needed to understand how online language teacher communities on Facebook specifically can be used as a professional source for language teachers using technology. As more language teachers are required to use technology for their teaching, it is vital to understand how they are actually capitalising upon them to learn about technology for teaching purposes.

Moreover, based on the literature, it does not seem too much of a leap to assume that online communities may be particularly useful during crisis situations such as the pandemic when in-person interactions are difficult. Since the outbreak of COVID-19, there seems to be some evidence that teachers turned to these online communities (e.g., Al-Jarf, 2021; Carpenter et al., 2021; Trust et al., 2020). Considering that the pandemic has been one of the first serious global pandemics since the existence of online communities on SNSs, there is still a shortage of studies investigating how they can be used by language teachers in times of a pandemic for professional purposes. To contribute to the current limited understanding of the topic, the study examined language teachers’ utilisation of a technology-focused language teacher community on Facebook during the pandemic, guided by the following main research question:

RQ: How are language teachers using a technology-focused language teacher community on Facebook during the COVID-19 pandemic?

## Methodology

### Data collection

Relevant studies on online teacher communities on SNSs have predominantly employed online observations, interviews, questionnaires, or a combination of these data collection methods. As indicated in Table 1, many studies have often been carried out over a short period of time and produced only snapshot views of them. Hence, unlike past studies, the current study was longitudinal in nature, adopting a mixed-methods research design.

**Table 1:** Overview of relevant studies on online teacher communities on SNSs

Study	Online platform	Main methods
Bissessar (2014)	Facebook	Interviews with group administrators (n = 4) and group members (n = 22)
Britt and Paulus (2016)	Twitter	Observations of education related tweets Interviews Archival documents
Carpenter et al. (2020)	Instagram	Questionnaire (n = 841)
Carpenter and Krutka (2015)	Twitter	Questionnaire (n = 494)
Curwood and Scott 2017	Twitter	Questionnaire (n = 64) Semi-structured interviews (n = 8) Observation of education related tweets (n = 530)
Goodyear et al. (2019)	Twitter	Observation of PE related tweets (n = 901) among 100 participants In-depth semi-structured interviews (n = 18)
Patahuddin and Logan (2018)	Facebook	Observation of Facebook responses to four posts about mathematics examples (n = 117)
Nilimarkka et al. (2021)	Facebook	Analysis of posts, comments, and reactions of a Facebook group (eight years) using API Participant observation
Rutherford (2010)	Facebook	Analysis of 187 discussion posts of a Facebook group
Schroeder et al. (2019)	Pinterest	Questionnaire (n = 117)
Wesely (2013)	Twitter	Participant observation of interactions among language teachers using specific hashtags Interviews (n = 9)
Staudt Willet and Carpenter (2020)	Reddit	Analysis of four different teaching-related subreddits (114,524 posts and 23,777 responses) for 12 months using API
Yildirim (2019)	Facebook	Observation of posts shared in a Facebook group for mathematics teachers (n = 2442) Interviews (n = 14)

#### Online observations

A public online community on Facebook for language teachers who are interested in technology was closely observed for eight months. It was created in May 2009 and has nearly 1000 members. Many of its members are language teachers who are residing in Japan, but since the community is open to the public, membership is not limited to only those living in Japan. It was selected as it met the following three criteria which were developed based on the research questions and existing literature. Firstly, the aim of the study was to understand what is happening in an online community for language teachers using technology, so the community needed to consist of members who fit this description. Secondly, considering the linguistic background of the present researcher, the main language for communication was to be English. Thirdly, the online community was to be found on Facebook since previous research has revealed that many teachers turn to Facebook groups for help with their teaching (e.g., Bissessar, 2014; Nilimarkka et al., 2021; Patahuddin & Logan, 2018; Rutherford, 2010; Yildirim, 2019).

The content and number of posts shared in the Facebook community and the number of members were manually recorded from December 2019 for a period of eight months.

#### Questionnaires and semi-structured interviews

The questionnaires and interviews were administered to understand the backgrounds and experiences of the community members (see Appendix 1 for further details). The initial questionnaire was distributed online in the observed community in June 2020, and in total, 51 community members responded to it. All but one of the questionnaire respondents were living in Japan at the time when they filled out the questionnaire. Approximately 60 percent were female (n = 29), and the majority of the questionnaire respondents were from English-speaking countries, most notably from the U.S. (n = 22), and teaching at university level (n = 44), which seems to reflect the current demographic of the observed community. The respondents had varying levels of teaching experience, with five being early in-service teachers (i.e., less than five years of teaching experience), 16 being middle-level in-service teachers (i.e., 6–15 years of teaching experience), and 29 being experienced in-service teachers (i.e., 16 + years of teaching experience).

All the initial questionnaire respondents were invited to participate in the interviews, and an invitation email was sent to 15 participants who left their email addresses. Of the 15 participants, 13 responded to the invitation and volunteered to take part in the interviews. All 13 interviewees were foreign nationals who were teaching English in Japan. Considering the exploratory nature of the study, the interviews took a semi-structured format. The interviews were conducted online via Zoom between August 2020 and June 2021, and the average interview time was 61 min. After the interviews, all the interviewees were asked to fill out a short questionnaire in which they were asked about their experiences of participating in the online community and teaching practice, and all but one responded.

### Data analysis

Content analysis was employed as the main data analysis technique. An inductive approach was taken, meaning that the identified codes, categories, and themes derived directly from the data (Kyngäs, 2020). All the initial posts shared in the observed community were categorised, and common types of posts were identified. Other data obtained from the online observation and questionnaires were statistically analysed using Microsoft Excel. All the interviews were transcribed to a text file and read repeatedly to create a coding frame to identify relevant themes. After the data generated from each of the three data collection instruments were analysed separately, they were combined and interpreted thematically.

### Ethical considerations

Although the researcher informed the administrators of the community, the online community members were not informed about the research. Firstly, seeking informed consent from all members of a large Facebook community is unrealistic and close to impossible. Some scholars argue that using data from publicly open online communities without informed consent may be justified if the discussion topics are not particularly sensitive (e.g., McKee & Porter, 2009). Since the Facebook community which was observed is open to the public and the topics discussed in the community are mostly related to technology in language teaching and learning and not particularly sensitive, the observation was conducted without seeking informed consent. In addition, to minimise bias, the researcher did not post in the online community to explain about the study as it would likely have influenced the outcome of the posts shared in the community.

The direct and paraphrased quotes of the online posts were checked in a variety of search engines to see whether the original sources were traceable. For the questionnaires and interviews, informed consent was received. A detailed description of the aims of the study and the purpose for which the data would be used was stated at the very beginning of the questionnaire and interviews, and only those who agreed participated.

## Results

### Online observations

**Fig. 1:**
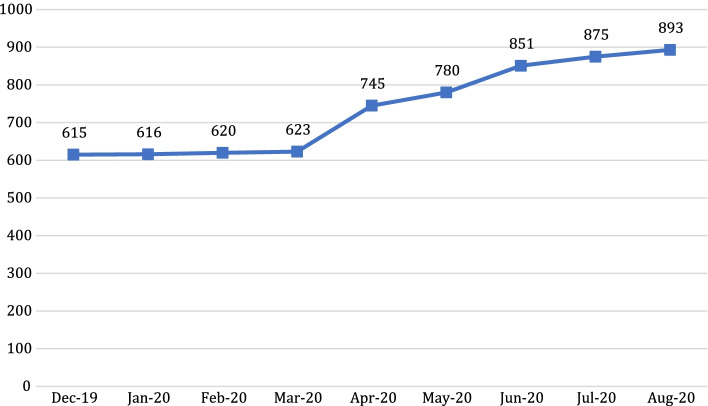
Number of community members per month (December 2019–August 2020)

As indicated in Fig. 1, there was a steady increase of the number of people joining the community during the eight-month period. On average, there was an increase of 31.1 members per month. There was a particularly large increase in the number of people joining the community in April 2020, when an additional 122 new members joined.

**Fig. 2:**
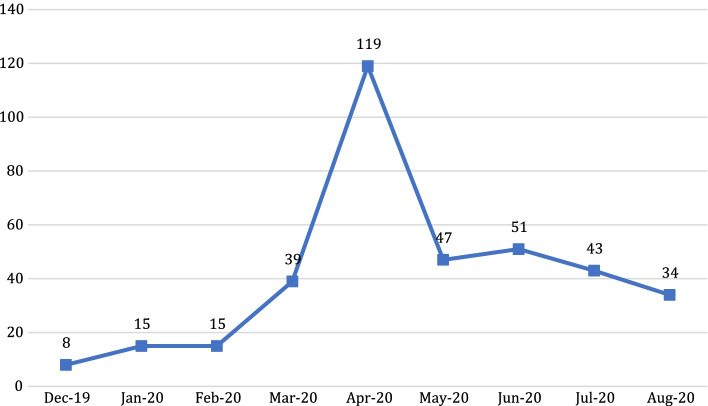
Number of posts per month (December 2019–August 2020)

In the eight-month period, there were a total of 340 posts shared in the observed community. As clearly indicated in Fig. 2, the month which had the biggest increase of posts was in April 2020: During the one-month period of March and April 2020, there was an additional 80 new posts. It should also be noted that there was a dramatic decrease in May 2020: There were 72 less posts than the previous month.

Of the 340 posts, 246 posts (72.4%) were about sharing information or resources and 94 posts (27.6%) were about asking for assistance. A content analysis of the 246 posts about sharing information or resources resulted in 11 main categories, which are summarised in Table 2. The most common type of posts about sharing information or resources was about upcoming events (30.1%) which were typically related to language, teaching, and/or technology. There were a few posts about online social events for language teachers offered by a different Facebook teacher community during the pandemic (i.e., twice in April 2020 and once in July 2020).

**Table 2:** Categorisation of posts about sharing information and resources (n = 246)

Types of posts	Count	Examples
Future events (e.g., conferences, seminars, lectures, social gatherings)	74	“Hi XXX here, apologies for posting. YYY is doing an online conference…”
Websites and online applications	32	“Perhaps a good way to clean up your audio for online teaching…”
Reading materials (e.g., Journals, books, blogs, listicles, newsletters, memes)	31	“We are delighted to announce our new journal entitled XXX.”
Videos (e.g., How-to videos)	28	“I hope no one minds that I drop this here. A video I made just today that helps users go over the more complex settings in ZOOM”
Personal remarks (e.g., advice, thoughts, rants)	22	“Teachers in Japan: read this before you start planning your online classes…”
Call for papers/chapters/books	22	“The call for papers for XXXX is open! Details in the link. Please share widely and join us. https://conference2020….”
News	9	“Looks like it is going to happen. Maybe Wednesday or Thursday. Supermarkets will be open, but since other shopping and public transportation will be limited or stopped, you might want to stock up for three weeks on what you need that you can't get locally…https://mainichi.jp/english/articles/….”
Admin related	9	“Apologies if you were affected by the most subtle bug ever on the server…”
Event related resources (e.g., photos, reflections)	9	“I hope you had a good experience. I enjoyed the sessions I attended and was really happy to reconnect with old friends and meet people in my field…”
Courses	4	“Hey everyone, I am an English teacher in Kyushu and I run a free course on Ed Tech tips. Please join if you are interested.”
Communities/online communities	4	“Hey, lots of us in Korea are having to teach online, as we hear are you all. Zoom seems to be what many are going to use. so, we’re teaching ourselves how to do that. We have an active Facebook self-help group. feel free to join…”
Other (random online image)	2	

As shown in Table 3, 11 categories were also identified from the posts about asking for assistance (n = 94). The most frequent type of posts was about asking for techno-pedagogical assistance (41.2%). That is, the members were mainly asking for advice on how to use certain technology for teaching and suggestions for resources and materials for teaching.

**Table 3:** Categorisation of posts about asking for assistance (n = 94)

Types of posts	Count	Examples
Asking for techno-pedagogical assistance	39	“Anybody have a powerpoint or instructions how to use zoom to teach a class online?”
Personal requests (e.g., surveys, polls)	15	“All I want for Christmas…are a few more responses for my doctoral research. If you're working in ESL/EFL at a Japanese university, kindly respond or share.”
Asking for volunteers	14	“Looking for a volunteer to moderate the chat in an “Intro to Quizlet” Zoom meeting tomorrow at 10:30 am!”
Admin-related	8	”Is the full program for the upcoming XXX conference [annual conference] going to be posted online soon? Or, is it already up somewhere? I can’t seem to find it.”
Asking for technical support	6	“I have had at least three students who couldn't use Microsoft Edge…Has anyone else encountered this issue?”
Asking for information about research and publication	4	“The changes I have been watching in public HS edtech and course delivery as this all is going on and I really want to write an article about it all when I am done. Is that a study? How does one write something like this up?”
Asking for opinions	3	“What do we think? https://www.theguardian.com/XXX…”
Asking to connect with a member/researcher	2	“Anyone attending the conference this weekend? I would love to connect with you.”
Asking for information about study abroad	1	“Regarding study abroad programs at your university/high school, can you briefly describe what steps your institution takes to limit the liability to the institution (and more importantly) teacher participants.”
Asking for examinations	1	“Hello, I was wondering if anyone has any information or experience regarding the GTEC Academic exam?”
Asking for conference recommendations	1	“Does anyone have some recommendations for virtual conferences?”

In three different time periods before the pandemic (i.e., December 2019–February 2020), the start of the pandemic (i.e., March 2020–May 2020), and during the pandemic (June 2020–August 2020), both types of posts about asking for assistance and sharing information or resources were found, though the breakdown of the subcategories differed (see Appendix 2 for more details). Before the pandemic, the most frequent type of post was about sharing information about Call for Papers (n = 10). At the start of the pandemic, the two main types of posts were about sharing information about future events (n = 27) and asking for techno-pedagogical assistance (n = 26). In the following three months, sharing information about future events was the most frequent type of post (n = 47).

### Questionnaires and semi-structured interviews

In the initial questionnaire, all but one (n = 50) indicated that they had been using technology in some way in their classes before the pandemic. Out of the 50 questionnaire respondents who claimed that they were using technology in their classes, 44 respondents (88%) stated that the ways in which they used technology changed due to the pandemic. Most of the respondents reported that they used video conferencing tools (e.g., Zoom, Google Meet), Learning Management Systems (LMSs) (e.g., Google Classroom, Moodle), or both to teach online. Two of the respondents claimed that the pandemic did not affect their teaching as they had already been teaching online prior to the pandemic.

Following the initial questionnaire, semi-structured interviews were conducted with 13 of the community members. It was found that all the interviewees were in two or more language teacher communities at the time of the interview. From the interviewees’ responses, the perceived positive and negative aspects of being in language teacher communities on Facebook during a pandemic were identified.

Firstly, a few interviewees commented on the convenient and accessible aspect of the online communities. For instance, the following comment shows how language teachers can easily learn through reading the posts shared in the communities in their spare time:

We, as teachers, want to read, we want to learn, we want to expand our knowledge base, but we don’t often have the time to do that. I think, being part of groups like this helps to streamline information a lot better, so if I only have a five-minute break between my classes, I hop on to Facebook and something pops up about something interesting going on in the tech world then that’s my five-minute knowledge boost for the day.

More than a third of the interviewees also indicated that they viewed the online communities as a virtual space to get professional or emotional support. One interviewee explained how she turned to the observed community if she had a question since she believed that “it’s almost guaranteed that someone can help you*.*” Some of the interviewees seemed to find the online communities to be particularly important during the pandemic, as indicated in the following comment made by a language teacher who joined a language teacher community on Facebook at the beginning of the pandemic:

In the beginning [of the pandemic], it [the online community] was kind of like counselling because everybody else was freaking out and stressed, because they had the pressure of learning new technology themselves… We all relate to each other, so in the beginning it was more like a counselling kind of thing with helping each other, and that was useful.

The majority of the interviewees (n = 12) reported that they were suddenly told that they needed to teach online despite not having any experience in teaching online prior to the pandemic. Several interviewees indicated that they joined language teacher communities on Facebook at the beginning of the pandemic. One interviewee claimed that she joined the observed community in April 2020 to connect with language teachers who had prior experience in teaching online as well as those dealing with similar problems:

Developing an online course takes a very long time, but we were asked to do it in a matter of weeks with no training… So, I was looking to get insights from not only people who had done this before but also who were in the same situation that I was in.

Although many interviewees reported on the positive aspects of being in language teacher communities on Facebook, a few did express some concerns. Several interviewees reported that they had occasionally witnessed or personally received harsh comments in the communities. One interviewee claimed that she disliked how a member from the online language teacher communities made rude comments to her when she posted a question in the community. In addition, a different interviewee indicated that he was reluctant to post in the language teacher communities on Facebook as he wanted to avoid arguments:

I don’t like getting drawn into these online discussions and debates because it just doesn’t seem like it’s worth anyone’s time, so that’s probably why I don’t actually post much.

Looking on the bright side, one interviewee even claimed that through her experience of being in language teacher communities on Facebook, it has made her realise the importance of online etiquette and safety and tries to teach her students about the importance of how they present themselves online.

In the post-interview questionnaire, which was distributed after the interviews, the interviewees were asked some questions to clarify the information obtained from the interviews. In response to the question asking whether or not their teaching practice had changed as a result of participating in the online communities, nine out of 12 interviewees indicated “yes.” As indicated in the following comment, the interviewee explained how she was able to incorporate what she had learnt from the online communities into her actual lessons:

I have been able to source more tools, techniques and insights from online communities and implemented it into my lesson.

In a similar vein, an interviewee who did not have much experience in teaching online before the pandemic claimed that participating in the online communities enabled her to learn more about online teaching and directly benefited on what she had learned from the communities:

I’ve learned more about online teaching and made use of some of the recommendations that I found in the groups.

On the other hand, it should also be noted that a small number of interviewees (n = 3) indicated that their teaching practice had not changed as a result of participating in the online communities. All three interviewees commonly reported in the questionnaire that they were “very confident” in using technology for teaching purposes, and one of them commented that he did not rely much on the online communities during the pandemic because he was able to fix most of his problems alone:

I ended up mostly troubleshooting things on my own. It’s not that I was disappointed in the community I found online, only that I utilized it less than I thought I would when I joined.

From his response, it is evident that not all online community members were heavily making use of the online communities for professional learning purposes.

## Discussion

As the primary objective of the study was to uncover how language teachers were utilising technology-focused online language teacher communities on Facebook during the pandemic, in the current section, the ways in which language teachers in the study were affected by the pandemic situation are first described, and subsequently, the main benefits and challenges of using online language teacher communities during the pandemic are outlined.

Echoing the findings from the study conducted by MacIntyre et al. (2020) which showed that the pandemic situation created various difficulties for language teachers, the current study illustrated that it was a challenging and stressful time for the language teachers who participated in the study as well. The findings clearly showed that as a result of the pandemic, many language teachers in Japan were suddenly compelled to change their way of teaching and use a video conferencing tool, such as Zoom and Google Meet, and a certain type of LMS, such as Google Classroom and Moodle, to teach online in lieu of face-to-face classes. Despite not having prior experience in using such technological tools, many teachers reported that their institutions offered little or no training or help when they were forced to switch to online teaching. For example, one interviewee whose native language was English reported that her institution held basic online training sessions about LMSs and video conferencing tools for faculty members during the pandemic, but since most of the sessions were often only offered in Japanese, she was not able to attend. In another instance, a different interviewee reported that she did not receive any assistance from her university because “nobody at the university knew how to.” These examples suggest that some institutions in Japan had failed to provide adequate support for their teachers during the pandemic, and the few institutions that did try to help seemed to have neglected to include the faculty members who did not speak Japanese. On top of that, it seemed that the pandemic had been particularly tough on part-time teachers teaching at multiple universities. The interviewee who was teaching at more than two institutions indicated that she had to learn how to use specific video conferencing tools and LMSs for each of the universities she was teaching at without barely any support from her workplace. Because of the pandemic, these non-Japanese foreign language teachers who already had a high risk of feeling isolated at the workplace due to language, cultural, and economic barriers appeared to be marginalised even further.

The study’s findings clearly indicated that technology-focused language teacher communities on Facebook brought about numerous professional benefits to language teachers who were suddenly forced to teach online during the pandemic. In line with relevant literature (e.g., Wesely, 2013), the observed Facebook community was providing language teachers with valuable learning opportunities. For instance, the content analysis of the posts revealed that the most frequent types of posts were about promoting events, courses, and resources (e.g., research articles, books, videos, websites, and online applications) which concerns with language, teaching, and technology; thus, indicating that the community offered a rich source of information for language teachers using technology for teaching purposes. Since the focus of the discussions in the observed online language teacher community was on technology, it is not entirely unexpected that the shared content covered a wide range of topics related to online teaching, such as tips on how teachers can incorporate Internet-based resources (e.g., Quizlet, DeepL) in their language learning classes to enhance a specific language skill and how they can use video-conferencing tools (e.g., Zoom, Google Meet) to promote student interaction online. As mentioned in the preceding section, one interviewee pointed out how language teachers can easily learn about “something interesting about the tech world” by utilising the online language teacher community. Moreover, from the post-interview questionnaire, it was clear that many of the interviewees believed that their general teaching practice had been affected from browsing these types of online posts, and some explicitly stated that they were implementing the ideas and suggestions about online teaching found in the online communities into their own classes. This shows that by reading the content shared in the communities, they were learning about how others were using technology in their classes, which, in turn, led them to experiment with new teaching ideas, tools, and materials obtained from the online communities.

A further advantage identified from the findings was the fact that language teachers were able to easily ask questions to language teachers who were experts in the field. The second most common type of post identified from the online observations was asking for techno-pedagogical questions, and although in less frequent occasions, online community members were also asking technical questions about how to use certain technology. In the interviews, several interviewees distinctly praised this aspect of being able to ask these types of questions, and one commented that “they are very useful and reassuring to know that they are there.” As teachers need to have adequate technical support in order to integrate technology successfully into their classes (Park & Son, 2009), it is important for language teachers to have a virtual space like the observed community where they can turn to professionals for help.

In addition, corroborating the findings from past studies (e.g., Patahuddin & Logan, 2018) which found that teachers were receiving emotional support through online teacher communities on Facebook, the current study showed that some of the community members turned to the Facebook communities to receive emotional support during the pandemic. For instance, one interviewee used the term “counselling” to describe one of the online communities she was a member of since she was able to ameliorate her stress by venting, reading humorous posts, including memes, images, and teaching episodes, shared by others and participating in events that would not otherwise be possible to take place during a pandemic. Moreover, it comes to little surprise that other interviewees also indicated that the communities had helped them feel less lonely during the pandemic as all 13 interviewees indicated that they were working temporarily from home during certain periods of the pandemic and their usual engagement with colleagues were limited due to pandemic-induced restrictions. As the content analysis of the posts revealed that there were several posts that were sharing information about online social gatherings held after working hours where language teachers were able to get together and socialise with other teachers teaching in similar situations, these types of events appeared to have helped those who were struggling to cope with professional isolation. Through engaging in the online discussions with other online community members on Facebook and participating in the promoted social gatherings, conferences, and webinars which were conducted beyond the original Facebook platform using video-conferencing tools such as Zoom, they were able to get through tough times together.

Another noteworthy finding from the study is that the observed online community served as a virtual information hub where non-Japanese speaking community members were able to access some information about the news and other resources about COVID-19 in English. This coincides with the findings from other studies which showed that pre-existing communities on SNSs played an important role in providing language minorities with up-to-date crisis-related information during emergency situations (e.g., Cho et al., 2013; Jang & Choi, 2020). In countries like Japan where natural disasters frequently occur, pre-existing communities on SNSs could potentially function as a useful and valuable crisis communication platform.

Whilst it is clear that being in language teacher communities on Facebook has numerous benefits, the negative aspects should not be overlooked. As indicated in the interview responses, not all community members had a positive experience in using language teacher communities on Facebook. Since Facebook users are required to use “the name they go by in everyday life” (Meta, 2022), it is likely that in language teacher communities on Facebook, there are fewer online ‘trolls’ who make harmful and abusive comments than other online platforms which allow users to be completely anonymous (Cruz et al., 2018). Nonetheless, as evident in the interviewees’ responses, language teachers on Facebook still have the risk of receiving harsh and negative comments, which may deter them from utilising the communities. As suggested by Goodyear et al. (2019), a possible solution may be to have strict moderators or facilitators who closely monitor the discussions in the community.

It is also worth mentioning that one teacher in the study was using her negative experience in the online community as a learning opportunity. It may be that having a negative experience on the online communities can be turned into a hands-on learning opportunity for teachers to develop their digital literacy skills. As Huack and Kurek (2017) point out, before language teachers can assist learners to use technology for learning purposes, they first need to become digitally literate themselves. Participating in language teacher communities on SNSs may provide the teachers with opportunities to develop their basic skills and knowledge on how to use SNSs and other technological resources, which could potentially help build their confidence and capacity to use them for teaching purposes.

### Limitations

Although efforts were made to ensure the quality of the research, the study was not without limitations. Since only one Facebook community was examined, the results obtained from the online observations may not represent what is happening in other language teacher communities formed on Facebook. As there have not been many longitudinal studies in relevant literature, it seemed more appropriate to conduct a thorough examination of a single language teacher community for an extended period of time rather than observing multiple communities for a shorter period of time. Moreover, since the observed community was set as a public group, the findings may not reflect those of private groups. It is worth exploring in the future to see whether the nature of discussions is different between the two types of groups. A further limitation is the low participation rate of the questionnaires and interviews. Although there were around 850 members in the observed Facebook community at the time the questionnaire responses were collected, only a small number of members participated in the initial questionnaire (n = 51), semi-structured interviews (n = 13), and post-interview questionnaire (n = 12). As lurkers (i.e., those who observe but do not participate) are a common feature of online communities (Popover & Fullwood, 2018), it was anticipated that the participation rates would be fairly low. Nonetheless, the overall results should be interpreted with caution as it is likely that the questionnaire and interview results only reflect a partial reality of those using the observed community.

## Conclusion

The current longitudinal study provides some insights into the realities of a language teacher community on Facebook during a pandemic. Language teachers in different regions of Japan were providing each other with professional and emotional support in the observed technology-focused language teacher community on Facebook: In particular, when in-person interactions were limited, they were able to support each other through sharing information, resources, and words of comfort and asking teaching-related questions. Without the help from their peers in the communities on Facebook, teachers with no or little experience in using technology for teaching purposes would have struggled even more. Even those who were experienced in using technology benefited from the social aspects of the communities as they were able to socialise with each other in the online social gatherings and related events, which, in turn, helped them overcome their feeling of loneliness and isolation. Although the past two years have witnessed a proliferation of research in crisis management in education (e.g., Back et al., 2021; Moorhouse et al., 2021), the field, especially with regards to language teacher education, is still in its infancy. The study’s findings thus contribute to a better understanding of how language teachers can rely upon online communities for support during uncertain times and how institutional administrators, policymakers, and other relevant stakeholders can assist language teachers in future crisis situations.

Although it is unclear how language teachers will use the online communities once the pandemic ends completely, they are likely to continuously play an important role in assisting teachers who use digital tools for teaching purposes even in the post-pandemic era. Of course, technology-focused language teacher communities on Facebook will not be a panacea for combatting all the problems that language teachers encounter when they use technology, but it is undeniable that they provide valuable support for teachers, especially when institutional support is lacking.

## Data Availability

The datasets used and analysed during the current study are available from the corresponding author on reasonable request.

## Appendix 1

See Table 4.

**Table 4:** Characteristics and details of the questionnaire respondents and interviewees

Characteristics	Count	
	Questionnaire (n = 51)	Interviews (n = 13)
**Sex**		
Female	29	7
Male	21	6
Prefer not to say	1	
**Age Group**		
20–29	4	1
30–39	10	3
40–49	23	7
50–59	9	1
60 +	5	1
**Nationality**		
American	22	6
British	4	2
Japanese	6	
Australian	5	2
Irish	2	1
Trinidadian and Tobagonian	2	1
Canadian	1	
British-Canadian	1	
Iranian	1	
Malaysian	3	1
Filipino	1	
Thai	1	
Turkish	1	
Indian	1	
**Place of residence**		
Japan	47	12
*Kanto*	*24*	*6*
* Kinki*	*9*	*2*
*Chubu*	*7*	*2*
*Shikoku*	*1*	
*Kyushu*	*3*	*3*
*Hokkaido*	*1*	
*Tohoku*	*1*	
* Chugoku*	*1*	
Thailand	2	
Malaysia	1	
Turkey	1	
**Years of teaching experience**		
Less than 1 year	1	1
1–5 years	4	
6–10 years	6	4
11–15 years	10	4
16–20 years	11	2
20–25 years	11	1
25 years+	7	1
Not specified	1	
**Teaching context**		
University	44	9
High school	4	1
Elementary school	1	1
Language school	1	1
Freelance	3	1

## Appendix 2

See Table 5.

**Table 5:** Types of posts before and during the pandemic

Period	Main category	Percentage (count)	Subcategory	Percentage (count)
Pre-pandemic (Dec 19–Feb 20)	Asking for assistance	32.4% (12)	Asking for techno-pedagogical assistance	33.3% (4)
			Personal requests	33.3% (4)
			Asking for techno-pedagogical assistance	8.3% (1)
			Asking for technical support	8.3% (1)
			Asking for information about study abroad	8.3% (1)
			Asking for volunteers	8.3% (1)
			Admin related	8.3% (1)
	Sharing information or resources	67.6% (25)	Call for papers/chapters/books	37.0%(10)
			Event-related resources	25.9%(7)
			Admin-related	11.1%(3)
			Websites and online applications	7.4%(2)
			News	7.4%(2)
			Personal remarks	3.7%(1)
Start of the pandemic (Mar–May 2020)	Asking for assistance	25.4% (44)	Asking for techno-pedagogical assistance	59.1% (26)
			Asking for volunteers	13.6% (6)
			Personal requests	9.1% (4)
			Admin related	4.5% (2)
			Asking for information about research and publication	4.5% (2)
			Asking for opinions	4.5% (2)
			Asking for technical support	2.3% (1)
			Asking for conference recommendations	2.3% (1)
	Sharing information and resources	74.6% (129)	Future events	20.9% (27)
			Websites and online applications	18.6% (24)
			Reading materials	17.1% (22)
			Videos	13.2% (17)
			Personal remarks	12.4% (16)
			News	4.7% (6)
			Admin related	4.7% (6)
			Courses	2.3% (3)
			Communities/online communities	2.3% (3)
			Call for papers/chapters/books	2.3% (3)
			Other	1.6% (2)
During the pandemic (Jun–Aug 2020)	Asking for information	29.2% (38)	Asking for techno-pedagogical assistance	23.7% (9)
			Asking for volunteers	18.4% (7)
			Personal requests	18.4% (7)
			Admin related	13.2% (5)
			Asking for technical support	10.5% (4)
			Asking for information about research and publication	5.3% (2)
			Asking to connect with a member	5.3% (2)
			Asking for information about exams	2.6% (1)
			Asking for opinions	2.6% (1)
	Sharing information and resources	70.8% (92)	Future events	51.1% (47)
			Videos	12.0% (11)
			Reading materials	9.8% (9)
			Call for papers/chapters/books	9.8% (9)
			Websites and online applications	6.5% (6)
			Personal remarks	5.4% (5)
			Event-related resources	2.2% (2)
			Courses	1.1% (1)
			News	1.1% (1)
			Communities/online communities	1.1% (1)

## Abbreviation

SNSs: Social networking sites
